# Safety and Potential Effect of Intrauterine Infusion of Autologous Adipose Tissue-Derived Regenerative Cells in Patients With Implantation Failure: A Pilot Study

**DOI:** 10.7759/cureus.57220

**Published:** 2024-03-29

**Authors:** Fusanori Yotsumoto, Kenichi Yoshikawa, Toyofumi Hirakawa, Daichi Urushiyama, Chihiro Kiyoshima, Hisatomi Arima, Shohta Kodama, Hiroaki Nishikawa, Shin’ichiro Yasunaga, Shingo Miyamoto

**Affiliations:** 1 Department of Obstetrics and Gynecology, Faculty of Medicine, Fukuoka University, Fukuoka, JPN; 2 Department of Preventive Medicine and Public Health, Faculty of Medicine, Fukuoka University, Fukuoka, JPN; 3 Department of Regenerative Medicine and Transplantation, Faculty of Medicine, Fukuoka University, Fukuoka, JPN; 4 Department of Cardiology, Fukuoka University Nishijin Hospital, Fukuoka, JPN; 5 Department of Biochemistry, Faculty of Medicine, Fukuoka University, Fukuoka, JPN; 6 Department of Obstetrics and Gynecology, Iwate Medical University, Iwate, JPN

**Keywords:** adipose-derived regenerative cell, adipose tissue-derived stem cell, infertility, endometrium, implantation failure

## Abstract

Background: Implantation failure due to thin endometrium has emerged as a major cause of infertility. In this study, we aimed to assess the safety and preliminary efficacy of adipose tissue-derived regenerative cells (ADRCs), a source of adipose-derived stem cells, in infertility patients with implantation failure.

Methods: Five infertile women with implantation failure despite artificial reproductive technology were enrolled in this study and treated with ADRCs via the intrauterine route. The primary outcome was the incidence of adverse events. Additional outcomes were endometrial thickness after ADRC treatment and pregnancy success after embryo transfer.

Results: There were no adverse events in any patient. There was no elevation of white blood cell count, C-reactive protein, or D-dimer levels. There was a significant difference in endometrial thickness in the secretory phase before versus after intrauterine transplantation of ADRCs (3.8 ± 1.3 mm versus 8.8 ± 2.8 mm, respectively; p<0.05). A gestational sac and fetal heartbeat were detected on transvaginal ultrasound in two of five patients.

Conclusion: Intrauterine infusion of autologous ADRCs is a simple and safe procedure that may ameliorate the endometrial microenvironment in infertile women with implantation failure.

## Introduction

Assisted reproductive technologies have contributed to infertility treatment [1]. Endometrial thickness plays a crucial role in embryo implantation and pregnancy achievement [2]. The thin endometrium is generally defined as less than 7 mm on the day of luteinizing hormone surge or human chorionic gonadotropin administration [3]. Implantation failure resulting from thin endometrium is a major cause of infertility; most infertile patients do not reach the minimum endometrial thickness in the luteal phase [3]. Several studies have investigated treatments for thin endometrium, including administration of exogenous estrogen, low-dose aspirin, sildenafil citrate, pentoxifylline, vitamin E, L-arginine, and cytokines; cell therapy with monocytes; and electroacupuncture and biofeedback therapy [4]. However, these treatments have not shown evidence-based effectiveness. Recent clinical trials have indicated that stem cell transplantation and platelet-rich plasma (PRP) have therapeutic effects [5]. Therefore, the development of regenerative medicine may improve thin endometrium in implantation failure.

Stem cells can renew their populations and differentiate into multiple cell lineages [6]. There are four main sources of stem cells: embryonic tissues, fetal tissues, adult tissues, and differentiated somatic cells after genetic reprogramming (called induced pluripotent stem cells) [7]. Stem cells have been used in preclinical and clinical trials, although the use of differentiated cells in tissue engineering is limited by the low quantity of harvested cells and their low proliferation potential during in vitro expansion. In clinical studies of regenerative medicine, bone marrow mesenchymal stem cells and endometrial stem cells have been used to improve the thin endometrium [8,9]. However, the wide use of regenerated cells to improve thin endometrium will require that the therapeutic agent have a reasonable cost.

Adipose tissue is a multifunctional organ containing various cell types, including mature adipocytes and the stromal vascular fraction (SVF) [10]. The SVF consists of adipose-derived stem cells, endothelial progenitor cells, pre-adipocytes, lymphocytes, mast cells, pericytes, and adipose-resident macrophages with repair and regenerative potential [11]. Adipose tissue-derived regenerative cells (ADRCs), which are almost equivalent to the SVF of adipose tissue prepared with the Celution system [12], mainly include adipose-derived stem cells in the uncultured condition. The use of ADRCs as a treatment for several diseases has been a recent focus of research in regenerative medicine [13,14]. ADRCs may be the most advantageous cells isolated from adipose tissue because of their abundance, subcutaneous location, and the less-invasive techniques used to collect them.

In a preclinical study, we demonstrated that intrauterine infusion of ADRCs as regenerative cells was valid in an experimental mouse model of thin endometrium [15]. Herein, we report the results of a pilot study designed to assess the safety and therapeutic potential of ADRCs in five infertility patients with implantation failure.

## Materials and methods

Study design

This prospective investigator-initiated clinical trial was undertaken as an open-label, single-arm, single-center study and was conducted at Fukuoka University Hospital, Fukuoka, Japan, in accordance with the principles of the Declaration of Helsinki. The study protocol was approved by the Institutional Review Board of Fukuoka University Hospital (approval number: 23), the Japanese Association for the Promotion of State of the Art in Medicine, and the Japanese Ministry of Health, Labour, and Welfare (registration number: PB7170015).

The primary outcome was the safety of intrauterine ADRC implantation. Secondary outcomes were endometrial thickness measured with transvaginal ultrasound before and after intrauterine ADRC implantation, confirmation of pregnancy with a pregnancy test, and detection of intrauterine gestational sac and fetus on transvaginal ultrasound after embryo or blastocyst transfer.

Patient selection (eligibility criteria)

Study-inclusion criteria were as follows: (1) diagnosis of infertility, (2) suspected embryo implantation failure, (3) need for artificial reproductive technology, (4) sufficient potential to maintain homeostasis (within three months before trial entry; white blood cells >3,000/μL; neutrophils >1,000/μL; platelets >100,000/μL; hemoglobin >10.0 g/dL; AST and ALT <100 IU/L; serum creatinine <1.0 mg/dL; total bilirubin <1.0 mg/dL; no diagnosis of cardiac dysfunction; D-dimer <1.0 μg/mL or >1.0 μg/mL with no deep vein thrombosis on ultrasound of lower extremity; PT <15 sec; and APTT 24-38 sec), (5) age ≥20 and ≤45 years, (6) oocytes and blastocysts vitrified after insemination by sperm from husband, and (7) voluntary agreement of patient and husband to participate in this clinical trial.

Exclusion criteria were as follows: (1) serious illness or suspicion of serious illness; (2) need for psychotic therapy or psychiatric medication; (3) poorly controlled diabetes; (4) active or poorly controlled serious infection; (5) disease that would worsen with pregnancy; (6) inflammatory disease or neoplastic endometrial lesion; (7) serious drug hypersensitivity; (8) presence of multiple malignancies or history of multiple malignancies in previous five years (excluding intraepithelial neoplasm); (9) use of oocytes and blastocysts other than patient’s own; (10) presence of malignancy or current chemotherapy, radiation therapy, or other cancer therapy; (11) serious disease, including cardiac disease, pulmonary disease, liver disease, kidney disease, hemorrhagic diathesis, sepsis, and hypertension; (12) use of anticoagulant or antiplatelet drugs or inhibitors of GP IIb/IIIa; (13) use of anticoagulant drug within one hour before harvesting of adipose tissues; (14) APTT elevation more than 1.8 times normal range; (15) active infection, including HIV, syphilis, and hepatitis B or C; (16) current pregnancy, nursing, or possible pregnancy; (17) treatment with other stem cell therapy; and (18) patients whose participation was deemed inappropriate by principal investigator or sub-investigators.

All participants underwent 12-lead electrocardiography, chest radiography, and respiratory function testing before liposuction to confirm the absence of abnormalities. Gynecological examination, transvaginal ultrasonography, and pelvic magnetic resonance imaging were performed to confirm the absence of intrauterine abnormalities before treatment. No concurrent use of regenerative medicine was permitted during the study.

Adipose tissue harvesting (liposuction)

Adipose tissue was obtained from the subcutaneum of patients’ buttocks with the tumescent liposuction method under general anesthesia. About 150-360 mL of adipose tissue was harvested and processed with a Celution® 800/CRS adipose tissue dissociation system (Cytori Therapeutics Inc., San Diego, CA), which is an investigational device that digests adipose tissue automatically under sterile conditions to isolate ADRCs according to the manufacturer's instructions. Briefly, the tissue was cleaned to remove the blood and remnants. Next, the aspirated adipose tissue was digested by adding Celase® GMP (Cytori Therapeutics Inc.), which contains highly purified collagenase and neutral protease enzymes, and incubated at 37 °C for 20 minutes. After that, the ADRCs were concentrated by centrifugation, washed to remove the Celase® reagent, extracted from the system, and prepared in a prescribed volume of 5 mL in lactated Ringer's solution. Cell viability and count were confirmed with an automated fluorescence cell counter (LUNA-FLTM; Logos Biosystems Inc., Anyang-si, Gyeonggi-do, South Korea) and with microscopic examination using the trypan blue exclusion test. The isolated ADRC suspension was mixed with 10% (v/v) dimethyl sulfoxide and divided into eight aliquots of 1.0-5.0 × 10^7^ cells (cell viability >70%), transferred to 4-ml cryotubes, and stored in liquid nitrogen within 1.5 to two hours after harvest.

ADRC preparation

Frozen ADRCs were quickly thawed in a 37°C shaking water bath and washed twice with 5 mL of m-HTF medium (No. 93444; Kitazato Corp., Shizuoka, Japan) by centrifugation at 1,500 rpm for five minutes at 4°C, and then suspended in 0.8 mL of m-HTF medium on the day of intrauterine ADRC infusion. The ADRC suspension was transferred to a 1-mL syringe connected to a 3.0-Fr Kitazato ET Catheter (No. ET-G3017ART-30; Kitazato Corp.) on ice.

Study protocol

**Figure 1:**
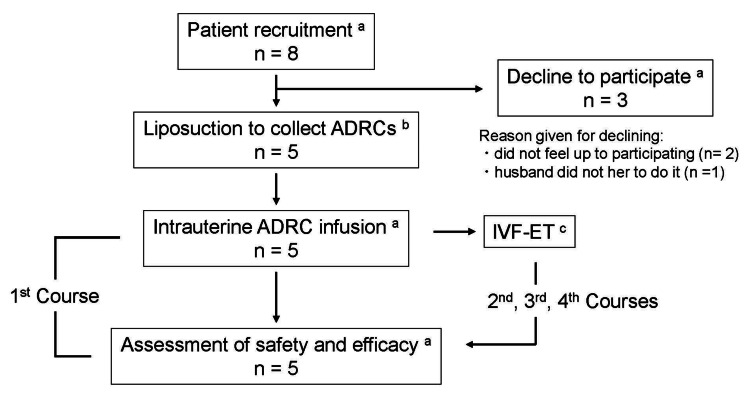
Flow chart of study design ^a^ Patient recruitment, intrauterine infusion of adipose tissue-derived regenerative cells (ADRCs), and assessment of treatment safety and efficacy were performed at Fukuoka University Hospital. ^b^ Liposuction to collect ADRCs was performed at the SOBAJIMA Clinic. ^c^ In vitro fertilization and embryo transfer (IVF-EF) was performed at Koga Fumitoshi Women’s Clinic in the second, third, and fourth courses of this study.

Figure 1 shows the study flow chart. Study entry and ADRC preparation were accomplished at the beginning of the study protocol. An intrauterine ADRC infusion was performed twice within seven days after the end of menstruation. Endometrial thickness was measured with transvaginal ultrasound twice on days three after intrauterine ADRC infusion and between days 10 and 17 of the menstrual cycle (putative luteal phase) at its greatest diameter perpendicular to the midsagittal plane in the fundal region. In the first course, the investigator prevented pregnancy in all patients with contraception to evaluate the safety of treatment. In the second, third, and fourth courses, all patients underwent embryo transfer (ET) after intrauterine ADRC infusion. Patients were placed in the lithotomy position. A catheter was passed through the cervical os to allow intrauterine injection of the ADRC suspension with 1.0 mL of hyaluronic acid (ARTZ Dispo 25 mg; Kaken Pharmaceutical Co., Ltd., Tokyo, Japan) and 0.2 mL of estradiol-17β (Ovahormone Depot 5 mg; ASKA Pharmaceutical Co., Ltd., Tokyo, Japan) diluted with 500 mL of normal saline. Patients remained in the lithotomy position for at least 15 minutes following the infusion to prevent leakage.

Safety evaluation

The safety of the intrauterine ADRC infusion was evaluated from the first infusion until the final ET. Adverse events were detected via vital signs, physical signs, gynecological examination, transvaginal sonography, complete blood counts, serum chemistry, D-dimer values, 12-lead electrocardiography, and chest radiographs. In each patient, vital signs, including body temperature, blood pressure, and pulse, were examined before and after intrauterine ADRC infusion. Adverse events, including patient-elicited, -solicited, and -volunteered symptoms, were graded according to the Common Terminology Criteria for Adverse Events v4.0 (CTCAE v4.0) set by the National Cancer Institute. Criteria for discontinuing this clinical study were defined as any adverse event or laboratory abnormality above grade 4 in the CTCAE v4.0 occurring immediately before the second course of intrauterine ADRC infusion. All adverse events were also evaluated after intrauterine ADRC infusion in the second, third, and fourth courses. Confirmation of menstruation after the fourth course was defined as the study's end in each patient.

Efficacy assessments

Endometrial thickness was measured with transvaginal ultrasonography in the luteal phase of the menstrual cycle immediately before the first course in each patient. Hormone replacement therapy was used in the previous cycle and first course to evaluate only the effect of intrauterine ADRC infusion on endometrial thickness. Endometrial thickness was also examined with transvaginal ultrasonography on days 10-17 of the menstrual cycle (3-10 days from the first ADRC infusion). Endometrial thickness in the previous course and first course were compared in each patient. In the second, third, and fourth courses, embryo or blastocyst transfer was performed after intrauterine ADRC infusion; chemical or clinical pregnancy was evaluated with a pregnancy test or transvaginal ultrasonography.

Statistical analysis

All values are expressed as the median (interquartile range). Wilcoxon rank sum tests were used to compare differences in median values (Prism 6; GraphPad Software Inc., San Diego, CA). P<0.05 was considered statistically significant.

## Results

Patient characteristics

Out of eight patients initially screened for eligibility, three patients were excluded from participating due to the patient’s and husband’s decline; five patients were included, and there were no patient dropouts in this study. Five women of reproductive age (two <40 years, three >40 years) with recurrent implantation failure and performance status 0 were recruited in this study (Table 1). The mean infertility period was 61.4 ± 16.9 months, and the number of ETs was 9.4 ± 2.1 at enrollment. All women had menstrual periods lasting one to three days, and menstrual volume seemed low. Patient 1 had undergone transcatheter uterine arterial embolization to treat cervical pregnancy in a previous gestation. All five cases were recognized as having severe implantation failure. All five patients completed the study according to the protocol; safety and efficacy were evaluated on an intent-to-treat basis.

**Table 1:** Clinical characteristics of patients ^†^Infertility period before registration in this study ^‡^Number of IVF-ET before registration in this study BMI: body mass index, IVF-ET: in vitro fertilization and embryo transfer

Patient no.	Age (years)	BMI (kg/m^2^)	Gravity/parity	Infertility period^†^ (months)	Number of IVF-ET^‡^
1	40	23.7	G2/P0	53	11
2	34	25.3	G1/P0	86	10
3	31	24.1	G2/P1	72	6
4	42	19.9	G0/P0	48	11
5	42	18.5	G4/P0	48	9

Safety

None of the patients reported abdominal pain, local pain, or fever at any time during the course of the intrauterine ADRC infusion. There were no elicited/solicited and patient-volunteered adverse events by intrauterine ADRC infusion. No alterations in body temperature, blood pressure, or pulse were found at any time during the infusion. Intrauterine ADRC infusion did not induce any direct or immediate adverse events. At the last observation date, no alterations in white blood cell count, C-reactive protein, or D-dimer level were seen in any patient (Table 2). No abnormal findings, including white blood cell count, C-reactive protein, D-dimer level, or gynecological examination findings, were found in the second, third, or fourth course. Regardless of the absence or presence of pregnancy, menstruation was confirmed after the final intrauterine ADRC infusion in all five patients. These results indicate that intrauterine ADRC infusion is safe.

**Table 2:** Adverse events in five patients with recurrent implantation failure None of the patients experienced uterine tenderness or other adverse events either pre- or post-treatment. CRP: C-reactive protein, NS: no significant difference between pre- and post-treatment values, Tx: treatment with adipose tissue-derived regenerative cells (ADRCs), WBC: white blood cell count, IQR: interquartile range

Laboratory data	Patient 1	Patient 2	Patient 3	Patient 4	Patient 5	Median (IQR)	p-value
WBC (10^3^/µL)							
Pre-Tx	6.7	8.5	8.2	8.3	8.4	8.3 (7.45-8.45)	NS
Post-Tx	7.6	9.1	6.3	7.0	7.8	7.6 (6.65-8.45)	NS
CRP (mg/dL)							
Pre-Tx	0.03	0.03	0.03	0.09	0.03	0.03 (0.03-0.06)	NS
Post-Tx	0.03	0.02	0.05	0.02	0.01	0.02 (0.015-0.04)	NS
D-dimer (µg/mL)							
Pre-Tx	0.6	0.8	0.6	0.8	0.5	0.6 (0.55-0.7)	NS
Post-Tx	0.8	0.6	0.5	0.6	0.8	0.6 (0.55-0.7)	NS

Efficacy

**Figure 2:**
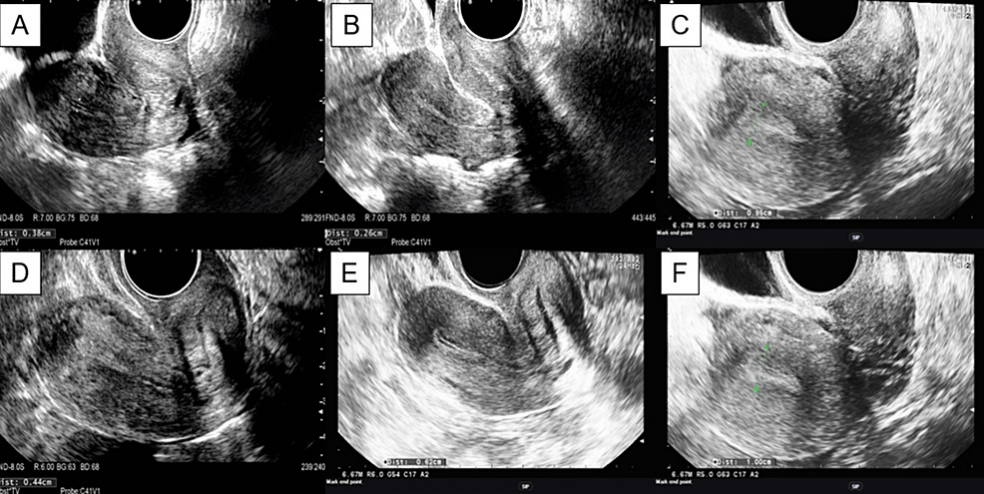
Endometrial thickness measured with transvaginal ultrasound in patient 3 (A) Thin endometrium (3.8 mm) in the secretory phase of the menstrual cycle before trial registration. (B) Endometrium directly after menstruation (2.6 mm) before ADRC treatment. (C) Uterine cavity filled with ADRC suspension with hyaluronic acid and estradiol-17β (9.5 mm). (D) Slightly thickened endometrium (4.4 mm) on the second day after intrauterine ADRC infusion. (E) Endometrium with a multilayered appearance in the late proliferative phase (6.2 mm). (F) Marked improvement of endometrial thickness in secretory phase after first ADRC infusion (10 mm). ADRC: adipose tissue-derived regenerative cell

Patient 3 had an extremely thin endometrium with rough morphology even in the secretory phase of the menstrual cycle (Figure 2A); the endometrium was almost as thin as immediately after menstruation (Figure 2B). Intrauterine ADRC infusion filled the uterine cavity (Figure 2C); no ADRC leak occurred after any infusion. Two days later, the endometrium looked slightly thicker (Figure 2D) than before the ADRC infusion, suggesting that hyaluronic acid is absorbed within a few days. The late proliferative-phase endometrium had a trilaminar appearance with a central thin line, a darker echolucent rim in the middle, and an echogenic basilar layer (Figure 2E). In the putative secretory phase (day 16 from the beginning of menstruation), the endometrium was 10 mm thick and appeared homogeneously hyperechoic (Figure 2F). A comparison of endometrial thickness in the secretory phase of the previous menstrual cycle with that in the putative luteal phase of the first course showed that intrauterine ADRC infusion induced a significant increase in endometrial thickness in all five patients (Table 3). However, there was no significant difference in endometrial thickness before and three days after intrauterine ADRC infusion (Table 3). In the second, third, and fourth courses, embryonic or blastocyst transfer was performed between days 22 and 30. Endometrial thickness was not greater than 7 mm in any patient at transfer. In six cycles (fourth course in patients 2 and 5, third and fourth courses in patients 3 and 4), human menopausal gonadotrophin was frequently administered after treatment with gonadotrophin-releasing hormone; patients 3 and 4 conceived clinically. In the nine other courses, estradiol was frequently administered; none of these patients conceived. Gestational sac and fetal heartbeat were confirmed with transvaginal ultrasound in cases 3 and 4; however, fetal heartbeat disappeared by eight or 10 weeks’ gestation (Table 4). These results suggest that intrauterine ADRC infusion may dramatically improve recurrent implantation failure, depending on the timing of administration and additional hormonal therapy.

**Table 3:** Endometrial thickness at the first course in five patients with recurrent implantation failure ^†^Total number of cells in two intrauterine ADRC infusions in the first course ^‡^Number of days after the first ADRC infusion ^§^p<0.05, significant difference between pre- and post-treatment values ADRC: adipose tissue-derived regenerative cells, Tx: treatment with ADRCs, IQR: interquartile range

Patient no.	Number of ADRCs (cells)^†^	Pre-Tx (mm)	Post-Tx1 (mm) (days)^‡^	Post-Tx2 (mm) (days)^‡^	Post-Tx2/pre-Tx
1	1.0 × 10^8^	4.9	4.8 (3 days)	12.0 (16 days)	2.45
2	5.8 × 10^7^	2.6	3.1 (3 days)	10.0 (14 days)	3.85
3	4.0 × 10^7^	3.9	3.9 (3 days)	10.0 (10 days)	2.56
4	5.0 × 10^7^	2.3	2.6 (3 days)	5.5 (12 days)	2.39
5	3.8 × 10^7^	5.1	5.8 (3 days)	6.4 (10 days)	1.26
Median (IQR)		3.9 (2.45-5.0)	3.9 (2.85-5.3)	10.0^§^ (5.95-11.0)	2.45 (1.795-3.205)

**Table 4:** Pregnancy outcomes of IVF-ET after intrauterine infusion of ADRCs in five patients with recurrent implantation failure CP: clinical pregnancy (identification of fetal heartbeat), IVF-ET: in vitro fertilization and embryo transfer, ND: not done, NPT: negative pregnancy test, ADRCs: adipose tissue-derived regenerative cells

Patient no.	Second course	Third course	Fourth course
1	NPT	NPT	NPT
2	NPT	NPT	NPT
3	NPT	NPT	CP
4	NPT	NPT	CP
5	ND	NPT	NPT

## Discussion

In this study, an intrauterine ADRC infusion was repeated eight times in patients with severe recurrent implantation failure. No adverse symptoms or signs were found during this study. On average, endometrial thickness increased 2.5-fold; three of five cases had thickness over 8 mm. In addition, two of five cases conceived a clinical pregnancy. This evidence indicates that intrauterine ADRC infusion is an easy, safe, and effective treatment for patients with severe recurrent implantation failure.

Intrauterine administration of various humoral factors, cell therapies, and mechanical procedures has been used to treat infertility in women with implantation failure [4,5]. Intrauterine administration of various substances is considered safe; the intrauterine ADRC infusion in this study was also considered a safe procedure. In this study, hyaluronic acid was added to ADRCs to prevent leakage from the uterine cavity. The hyaluronic acid appeared to be almost completely absorbed within a few days. Human oocytes are surrounded by hyaluronic acid, which acts as a natural selector of spermatozoa [16]. In addition, hyaluronic acid is produced by oocytes and embryos and contributes to pre-implantation embryonic development and implantation [16]. Hyaluronic acid, which is part of the extracellular matrix, is also bound to adipose-derived stem cells through CD44 and Toll-like receptors 2 and 4, which are expressed on cell membranes [17,18]. After adipose-derived stem cells bind to hyaluronic acid, they differentiate into a variety of cells and promote angiogenesis and tissue regeneration [17,18]. Residual hyaluronic acid in the uterine cavity may be beneficial in patients with recurrent implantation failure. Intrauterine administration of ADRCs plus hyaluronic acid and estradiol is considered safe and effective in treating implantation failure in patients with thin endometrium.

The patients with more than 8 mm of endometrial thickness after intrauterine ADRC infusion were 31, 34, and 40 years of age, whereas both patients with less than 7 mm thickness were 42 years of age. In a previous study, intrauterine infusion of autologous menstrual blood-derived stromal cells (1 × 106 cells infused) induced more than 8 mm of endometrial thickness in only one of seven patients under age 40 with severe Asherman syndrome [8]. Many studies have evaluated PRP as a treatment for implantation failure, all in patients below 40 years of age with refractory endometrium of approximately 7 mm thickness [19,20]. In patients over 40 years of age or with less than 5 mm of endometrial thickness, however, PRP infusion might not be effective. ADRCs have possible advantages over other regenerative materials, depending on the patient’s age and the number of administered cells [21]. It is plausible that the number of infused ADRCs should be altered, depending on patient age, endometrial thickness, and other factors.

In a study of 15 patients with refractory Asherman syndrome, a mean of 124.39 × 106 bone marrow-derived stem cells were delivered into the spiral arterioles by catheterization; cell therapy increased endometrial thickness for a few months, with a return to initial levels at six months [9]. Even with intra-arterial infusions of stem cells, the effects of cell therapy were not persistent. Likewise, intrauterine infusion of menstrual blood-derived stromal cells increased endometrial thickness for only a few months [8]. Endometrium evaluation and ET were performed two days after the intrauterine PRP infusion [8]. In this study, endometrial thickness at 14 to 17 days decreased by 22 to 30 days after intrauterine ADRC infusion. It is conceivable that most stem cells infused disappear by the next menstruation and that adipose-derived stem cells in intrauterine ADRCs rarely transform into endometrial stem cells in the uterine cavity. It is plausible that the effectiveness of infused ADRCs may continue after several days.

In addition to a thin endometrium, recurrent pregnancy loss and chronic endometritis may cause recurrent implantation failure. Abnormalities in the immune system, including an imbalance in T-helper lymphocytes (TH1 and TH2), a decrease in uterine natural killer cells, and the presence of antiphospholipid antibodies, are reported causes of recurrent pregnancy loss [22]. Bone marrow-derived or adipose tissue-derived stem cells induce anti-inflammatory cytokines and suppress pro-inflammatory cytokines in vivo and in vitro [23-25]. Natural killer cells are thought to differentiate from adipose tissue-derived stem cells [26]. The major cause of chronic endometritis is a chronic microbial infection characterized by persistent inflammation of the endometrial lining [27,28]. Adipose-derived stem cells exert antimicrobial effects through direct and indirect mechanisms [29,30]. Intrauterine ADRC infusion may allow patients with implantation failure resulting from recurrent pregnancy loss and chronic endometritis to have embryo implantation into a robust endometrium.

Limitation

Before establishing intrauterine ADRC infusion as a treatment for implantation failure, there are several issues to resolve: (1) What is the best interval between ADRC infusion and embryo or blastocyst transfer? (2) Which hormones should be combined with the ADRC infusion? (3) How many ADRCs should be infused, and does this depend on patient age or endometrial thickness? In addition, pre-implantation genetic testing for aneuploidy should be performed to check egg quality. Models of ultrasound examinations may have affected the study results because measurement methods for the endometrial thickness did not meet the criteria of the standard AIUM and ISUOG guidelines. Further large clinical trials should be performed on intrauterine ADRC infusions in the treatment of implantation failure.

## Conclusions

Autologous ADRC infusion is relatively inexpensive and is available for medical treatment. Intrauterine infusion is a simple and safe procedure. In this study, intrauterine ADRC infusion induced a marked increase in endometrial thickness and led to clinical pregnancy in two of five patients, suggesting that this treatment may be effective for patients with implantation failure.
